# Biomechanical Analysis of Bone Graft Treated With Pasteurization or Cryotherapy Using Liquid Nitrogen: A Study Using Sheep Bone Model

**DOI:** 10.7759/cureus.31780

**Published:** 2022-11-22

**Authors:** Nik Alyani Nik Abdul Adel, Kamarul Ariffin Khalid, Frederick Chin Khang Yee

**Affiliations:** 1 Department of Orthopaedics, Traumatology & Rehabilitation, International Islamic University Malaysia, Kuantan, MYS

**Keywords:** cryotherapy, pasteurization, liquid nitrogen, sterilized autologous bone graft, limb reconstruction surgery, osteosarcoma

## Abstract

Osteosarcoma is a common primary malignancy of the bone. Osteosarcoma prognosis improves tremendously when chemotherapy is given in adjunct to surgical intervention. Limb reconstruction with sterilized autologous bone graft stabilized by orthopaedics implants has become a choice of treatment, but the biomechanical property of the bone treated with such sterilization method is a point of concern as a graft with inferior biomechanical property might lead to graft failure. This study compares the biomechanical properties of the bones treated with cryotherapy using liquid nitrogen and pasteurization in the form of four-point bending compression tests. Six sheep tibia bones were used for each group of treatment with one control group where no treatment was given. In the four-point bending test, osteotomy was performed at the tibia; the proximal tibia was treated with liquid nitrogen and pasteurization respectively. The treated bone is then reconstructed with the corresponding distal bone with locking plate and screws (Jiangsu Trauhiu Medical Instrument Co. Ltd., Changzhou, China). The four-point bending test was performed with an Electropulse® E3000 universal testing machine (Instron®, High Wycombe, United Kingdom) and results in the form of maximum compressive load, stress, and strain were collected. Photographic analysis of the fracture location and pattern were documented and analysed. We found that both methods of sterilization did not alter the biomechanical properties of the treated bone to the extent of statistical significance in comparison with the control group. However, other factors such as histological changes post treatment, equipment availability, and long-term outcome must be considered.

## Introduction

Osteosarcoma is the most common primary malignancy of the bone, where malignant mesenchymal cells produce osteoid and/or immature bones [1]; and the most frequently seen non-haematopoietic, primary solitary malignancy of bone [2]. Demonstrating a bimodal age distribution, the incidence of osteosarcoma has been reported to be 4.4 per million population in the age group of 0-24 years and 4.2 per million population in the age group of 60-85 years [3].

In Malaysia, a study done at University Malaya Medical Centre from 1997 to 2011 shows 128 cases of osteosarcoma treated in 14 years [4], and a study at Hospital Universiti Sains Malaysia was able to retrieve 127 patients treated there from 1995 to 2007 [5]. Although this figure is not representative of the country, it still is a sizable figure and, hence, it can be said that this disease has a significant impact on the Malaysian population.

Treatment of osteosarcoma has improved over the years. Presently, primary chemotherapy, surgical intervention, and adjuvant chemotherapy are the most common treatment modalities for osteosarcoma [6,7]. Prior to the advent of chemotherapy, osteosarcoma was a fatal disease with patients surviving only a few months with metastasis at diagnosis. Chemotherapy increases the six-year survival rate from 11% to 61% [8,9].

Surgical excision of the osteosarcoma remains critical in obtaining remission and improving the survival rate. In the past, it was believed that amputation is the definite treatment for osteosarcoma. However, it caused further physical and psychological trauma to the ill patient. With the introduction of various reconstructive surgery options such as autografts, allografts, and endoprosthesis, less destructive surgical methods were available, leading to a more fruitful life and better functional outcome, without compromising long-term survival rate [7,10]. Up to 95% of patients with osteosarcoma can be treated with limb-sparing surgery at major centres specializing in musculoskeletal oncology [11].

Studies show that about 85% of high-grade appendicular osteosarcoma cases can be successfully resected and reconstructed with preservation of the affected limb and its function where this did not increase the rate of recurrence, provided that negative oncology surgical margins surrounding the tumour had been resected [12]. With adjuvant chemotherapy, limb-preserving surgery is able to provide a similar outcome and better functional outcome, previously thought to be only possible in limb amputation and this is the current mainstay of treatment in osteosarcoma [13-15].

Endoprosthetic reconstruction is the preferred method as it offers a certain degree of customization and can be adjusted to match the anatomical requirement of the patient individually. However, complications were encountered with this method such as loosening, breakage, and wear during long-term follow-ups [16,17]. Implant survivorship is over 80% at five years and drops to 60% in ten years; in a study in Britain, 42% of patients with endoprosthetic implants required revision within 10 years where 51% were due to mechanical failure and 33% were due to infection [18,19]. Endoprosthesis is expensive and requires speciality in implant support and peripheries, presenting a roadblock in obtaining a suitable implant at an acceptable cost to the patient.

Biological reconstruction provides a biological union between the host and the transplanted bone (allograft) or autograft-treated bone, and this allows for a stronger reconstruction. Organ donation rates vary across the world and some countries have lower deceased organ donation rates. In 2010, Myanmar had only 0.02 donations per million population, followed by Malaysia (0.48), Guatemala (0.52), Bulgaria (1.14), and Thailand (1.26) [20]. In allograft also, there are concerns about immunologic reactions, the transmission of diseases, and religious and social dissatisfaction in some regions [21]. As bone donation is not widely accepted in some countries, allografts are not readily available in this setting, and paired with the difficulty in getting a suitable graft fitting for the need of a specific patient and some other factors; it is not a ready option for many patients in some countries.

Autograft, hence, presents itself as a viable method. Bone autograft is a technique in reusing the diseased bone that has been excised and subjected to various treatments to ensure total eradication of bone tumours before being re-implanted into the defective area with significantly reduced cost, making the treatment option more accessible to patients. The treated bone graft is reconstructed to the patient’s limb usually with plate osteosynthesis or sometimes with an intramedullary device. Studies have shown promising results in using autograft in patients suffering from osteosarcoma. Chen et al. reported a functional recovery rate of 80% with an average of 40 months follow-up [22], while Bohm, et al. reported a 92% functional recovery rate with an average of 62 months follow-up [23]. Other benefits of autologous bone grafting include near anatomical fit, low risk of infection, no donor site morbidity, and lower risk of immunological reaction.

There are four common sterilization techniques used in current clinical settings: autoclave gamma irradiation, autoclaving, pasteurization, and cryotherapy [21]. All the methods are used to prepare autologous bone grafts by eradicating tumour cells in the bone, providing a disease-free component for osteosynthesis. However, as each method eradicates the cells in different manners, further study needs to be performed to review the effect of each method on the structural viability of the graft. Several studies have highlighted the good outcome of sterilized autologous bone grafts [7,8,21]. However, to date, only a few studies have been done to determine the biomechanical properties of sterilized autologous bone grafts [11,24,25], and no study has been done to compare sterilized autologous bone grafts reconstructed with implants.

The main objective of this study is to determine the superior method of sterilization in terms of immediate biomechanical properties of bones that are treated with cryotherapy using liquid nitrogen and pasteurisation, which are reconstructed with plate osteosynthesis. As the patient is expected to bear weight on the reconstructed limb, the mechanical strength of the treated bone is crucial to lowering the risk of failure. Specific objectives include comparing the biomechanical properties namely maximum compressive load, stress, and strain in a four-point bending test and observing the pattern of failure of the sample tested.

## Materials and methods

This is an experimental study using tibia bone of the common sheep,* Ovis aries*. The sheep tibia was selected as the study subject because of its anatomy and bone strength, which are almost identical to that of the human tibia bone. This minimizes the variation of bone properties. The animals were sourced from an animal farm that provides dairy meat. Animals used in the study were scheduled to be slaughtered for consumption. The animal bones were acquired after the animal is slaughtered. As no live animals were used, there is no conflict in the ethical ground of the study. This study has been approved by the Kulliyyah of Medicine Research Committee of International Islamic University, Malaysia (approval number: IIUM/305/20/4/1/17).

The sheep tibia bones were divided into three groups based on the sterilisation technique, namely cryotherapy, pasteurisation, and a control group where no treatment was given. Each of the groups consisted of six samples. The bone preparation was performed, namely osteotomized sheep tibia bone placed with an implant for four-point bending testing. The sample size calculation method used was the resource equation method described by Charan et al., where a sample size ranging from 10 to 20 using the formula of the total number of animals minus the total number of groups is deemed adequate [26].

**Figure 1:**
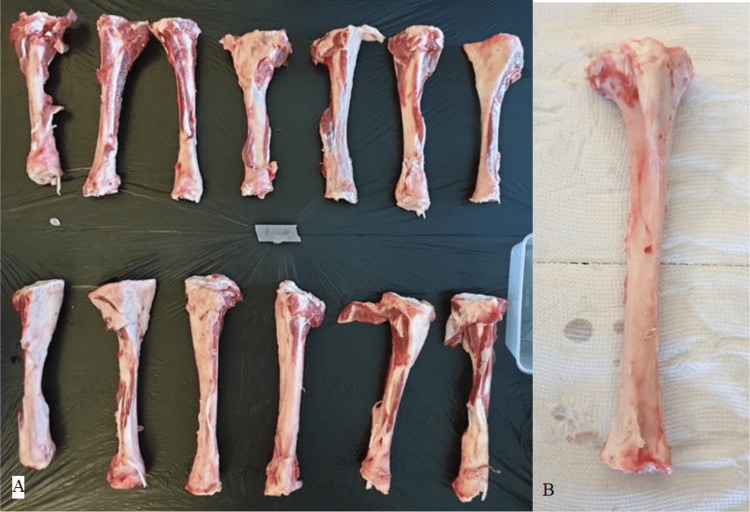
(A) Freshly harvested bones with muscles and tissue still attached; (B) Sheep tibia bone with tendons, muscle and soft tissues stripped.

The tibia of the sheep was taken immediately after the slaughtering process was done (Figure 1). The tibia was then stripped of soft tissue and wrapped with salted gauze for fixation purposes. Only similar diameters of the bones were used for this study. Then the tibia was covered with waterproof plastic wrap. To maintain the freshness of the bones, they were transported in a freezer box filled with ice and stored in a freezer capable of storing the bones at -80 degrees Celsius. Bones were kept at -80 degrees Celsius until their use to preserve their property.

**Figure 2:**
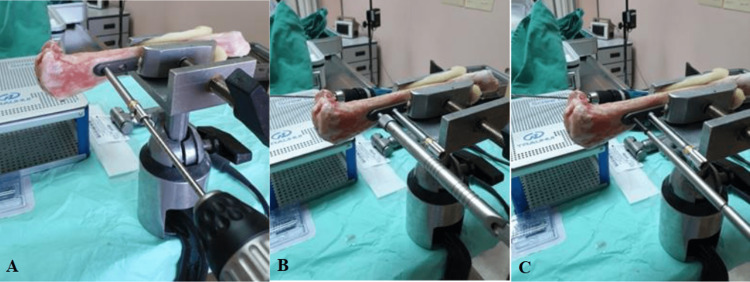
Plate osteosynthesis reconstruction of the sheep tibia bone is performed in steps as it would be done in a setting with real patient: (A) Dual cortex drilling of the bone with a locking drill guide; (B) Depth measurement to determine screw length; (C) Appropriate length screw insertion. The bone is protected in the clamp with foam padding to prevent focal clamping pressure.

Bones were thawed for 24 hours before the experiment was done. Plate osteosynthesis was performed first on all the sheep tibia using a titanium tibia straight plate with six holes (Jiangsu Trauhiu Medical Instrument Co. Ltd., Changzhou, China) and locking screws with a diameter size of five mm (Jiangsu Trauhiu Medical Instrument Co. Ltd) (Figure 2). These plates were applied on the same surface for all bones and three screws were inserted at each end of the osteotomy. Horizontal bone osteotomy was performed 10 cm from the proximal end of the tibia using Hall® PowerPro® Electric II Oscillator saw (Livantec Corporation, Florida, United States) with water cooling using 0.9% sodium chloride intravenous solution to reduce the risk of thermal necrosis during the process. This orthopaedic implant is commonly used in hospital settings to stabilize the midshaft tibia fracture and it is a type of tibial variable angle locking plate. The proximal part of the sheep tibia was marked and labelled to its distal part. The proximal part was removed from the plate by taking out its screws and ready for treatment based on the groups. 

**Figure 3:**
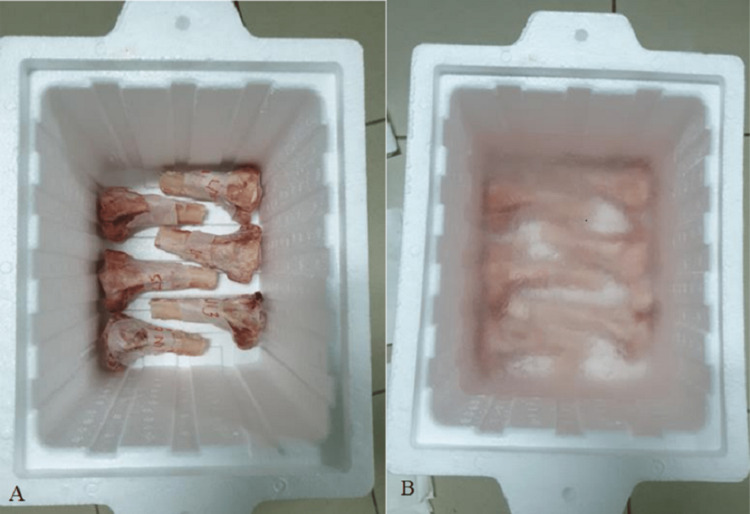
(A) Proximal tibia bone end with individual labelling contained in polystyrene box suitable for liquid nitrogen experiment; (B) Treatment of bones in liquid nitrogen.

When the bones were ready, they were treated using two methods. In the cryotherapy group, the prepared samples were put into medical-grade liquid nitrogen at a temperature of -196 degrees Celsius (Figure 3). The bone samples were placed in liquid nitrogen for 20 minutes, then stored at room temperature for 15 minutes, and finally stored in physiological saline for 15 minutes before being tested for biomechanical testing. This preparation protocol is performed in accordance with the technique used for bone grafts treated with liquid nitrogen as described in previous studies performed by Tsuchiya et al. and Yamamoto et al. [25,27]. 

**Figure 4:**
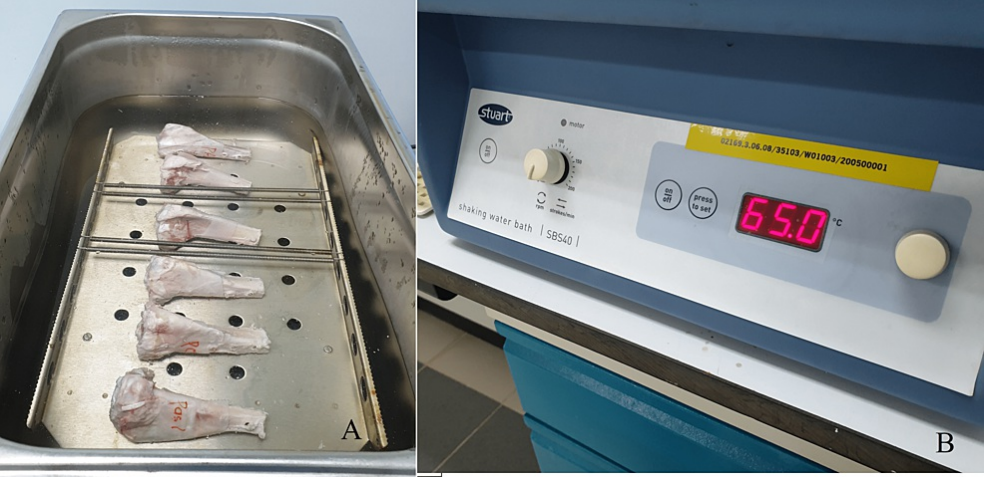
(A) Stuart SBS40 water bath used to pasteurize the specimen; (B) Temperature regulated at 65 degree Celcius.

For the pasteurisation study group, test samples were placed in a water bath machine (Stuart SBS40; Antylia Scientific, Vernon Hills, Illinois, United States) with 0.9% intravenous sodium chloride solution used (Figure 4A). The homeothermic heater was pre-set to 65 degrees Celsius and maintained at this temperature (Figure 4B). The proximal part of the tibia was then immersed in a water bath of 0.9% sodium chloride solution for 30 minutes. This protocol is based on previous studies in pasteurisation described by Manabe et al. [21]. No treatment was given to the bones in the control group. They were soaked in 0.9% sodium chloride solution at room temperature for 20 minutes before being tested.

All of these proximal tibia sections were then aligned with their marked distal part and screws were reinserted back over the proximal tibia to reconstruct the tibia. Bone samples that were reconstructed with plate underwent biomechanical testing using the Electropulse® E3000 universal testing machine (Instron®, High Wycombe, United Kingdom). A four-point bending test was performed to test the reconstructed sheep tibia bone with implants.

**Figure 5:**
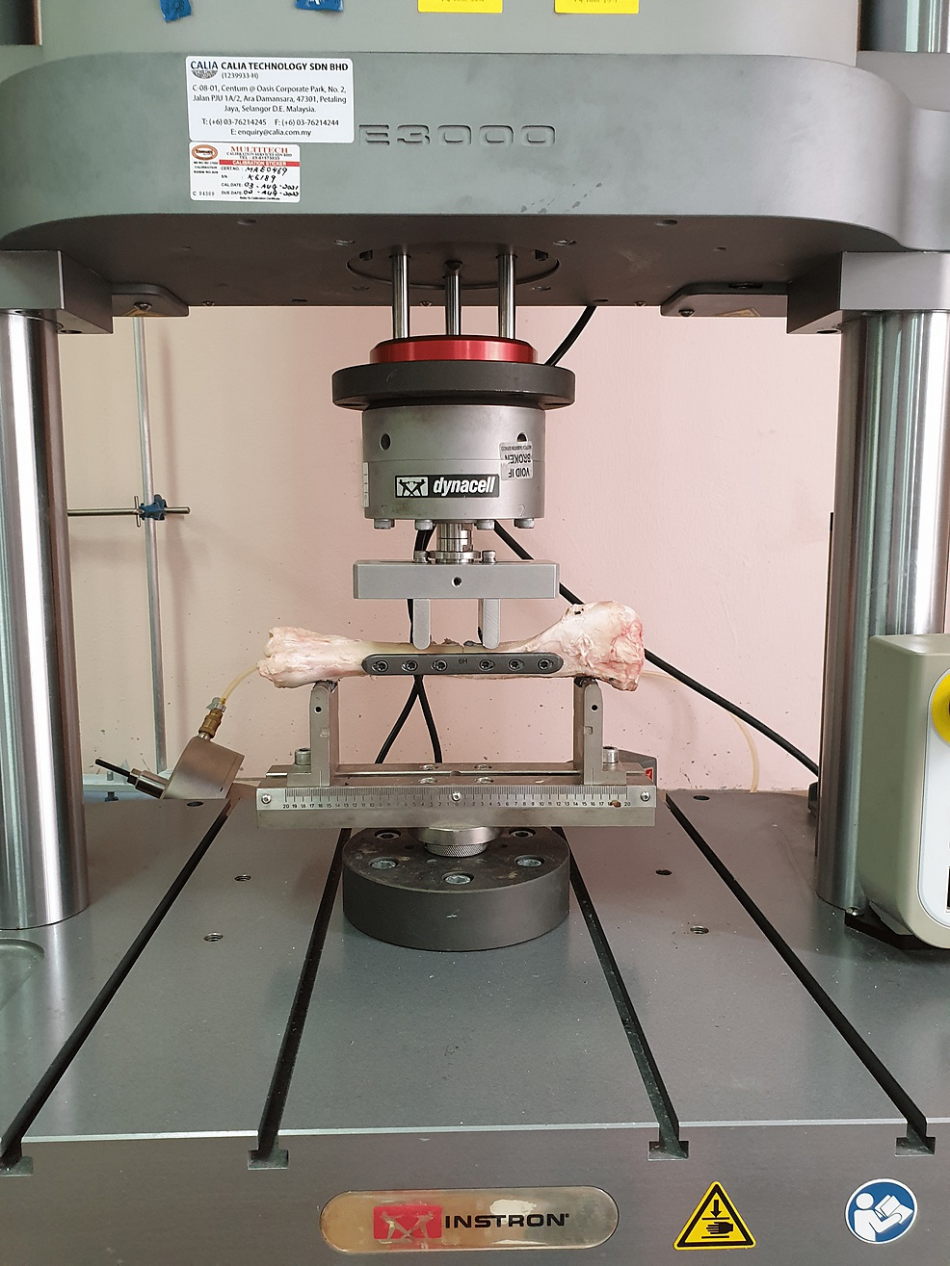
The four-point bending compression testing of the reconstructed sheep bone using the testing machine.

The reconstructed bone was placed horizontally on a four-point bending test jig and each bone was placed in the same orientation on the jig to standardize the study (Figure 5). Preloading of the bone was done by lowering the top points until they were just touching the bone. The bending test was then commenced by applying force from the top at a fixed rate of 50 Newtons per second (N/sec). The bending pressure is then applied in three N/sec increments. The load required to achieve the defect was documented on the computer system connected to the universal testing machine and the bones were tested until failure. Maximum compression load (Newton (N)), compression stress (Mega Pascal (MPa)), and compressive strain (mm) were calculated by the system. The location, modality, and pattern of fracture were photographed and documented. Modes and points of failure were photographed and compared. The parameters obtained were maximum compressive load, compressive stress, and compressive strain.

All data analyses were computed using IBM SPSS Statistics for Windows, Version 21.0 (Released 2012; IBM Corp., Armonk, New York, United States), consisting of paired-sample t-test to evaluate the significant difference between the treatment compared with the control group, and an independent sample t-test was used to determine the difference between the two treatments. In addition, one-way ANOVA was calculated to see the homogeneity between and within the treatment and control groups based on the parameter used. Comparative tests were used at the alpha level of probability (p <0.05) to determine significant differences between the means.

## Results

A total of 18 sheep tibias were used in this study, divided into three groups according to the type of treatment: cryotherapy, pasteurisation, and control group (no treatment). In the four-point bending test, the results showed mean maximum compressive load for sheep tibia treated with pasteurisation was 2263.667 (±293.42) N, which was slightly higher than the control group (2216.598N, ± 413.116) and cryotherapy group (2130.049N ± 540.03). In compressive stress, the pasteurisation group (14.411MPa ± 1.868) again showed a higher value than cryotherapy (13.560MPa ± 3.438) but slightly lower than the control group (16.40MPa ± 7.714). Meanwhile, in compression strain, the pasteurisation group (mean of 8.900mm ± 3.716) had a lower value than the control group (7.556mm ± 3.512) and the cryotherapy group (8.028mm ±3.268) (Table 1).

**Table 1:** Descriptive profile of four-point bending test on sheep tibias in three test groups.

No.	Treatment	Parameters	N	Mean	Median	SD
1	Control	Max. Compressive Load (N)	6	2216.598	2030.940	413.116
		Compressive Stress (MPa)		16.400	12.929	7.714
		Compressive Strain (mm)		6.416	7.556	3.512
2	Cryotherapy	Max. Compressive Load (N)	6	2130.049	2124.045	540.033
		Compressive Stress (MPa)		13.560	13.522	3.438
		Compressive Strain (mm)		7.970	7.858	0.843
3	Pasteurisation	Max. Compressive Load (N)	6	2263.667	2245.756	293.415
		Compressive Stress (MPa)		14.411	14.297	1.868
		Compressive Strain (mm)		9.669	8.900	3.716

There is no significant difference in maximum compressive load, compressive stress, and strain when comparing the cryotherapy group with the control group (p-value of 0.1, 0.11 and 0.728, respectively). Similarly, there was no significant difference in compressive load, compressive stress, and compressive strain when comparing the pasteurization group with the control group (p-value of 0.838, 0.55 and 0.065, respectively). When comparing the pasteurization group with the cryotherapy group, no significant difference was noted in all three biomechanical tests (p-value of 0.578, 0.578 and 0.322 for each test, respectively) (Table 2).

**Table 2:** T-test of mean difference of four-point bending test of the sheep tibial bone according to their treatment technique.

Test group	Test Parameter	df	4-Point Bending Test Data Difference	Std. Error Mean	t-value	p-value	Test group
			Mean (MD)	SD			
Control - Cryotherapy	Max. Compressive Load (N)	5	86.548	742.287	303.037	0.286	0.787
	Compressive Stress (MPa)		2.830	8.689	3.547	0.798	0.461
	Compressive Strain (mm)		-1.554	3.666	1.497	-1.038	0.347
Control - Pasteurisation	Max. Compressive Load (N)	5	-47.070	534.995	218.411	-0.216	0.838
	Compressive Stress (MPa)		1.979	7.577	3.093	.640	0.550
	Compressive Strain (mm)		-3.254	3.373	1.377	-2.363	0.065
Cryotherapy - Pasteurisation	Max Compressive Load (N)	5	-133.618	549.956	224.519	-0.595	0.578
	Compressive Stress (MPa)		-0.851	3.501	1.429	-0.595	0.578
	Compressive Strain (mm)		-1.700	3.788	1.547	-1.099	0.322

In conclusion, although bone treated with cryotherapy and pasteurisation showed a reduction in maximum compression load, compressive stress, and strain, except for maximum compressive load in the pasteurisation group, in comparison to the control group in the four-point bending test, it was not statistically significant. Hence, it can be concluded that bones treated with both cryotherapy and pasteurization show comparable biomechanical properties as the control group.

Tukey posthoc using ANOVA testing also revealed no significant differences between the parameters of analysis using cryotherapy as a treatment for sheep tibia reconstructed with plate osteosynthesis, compared with the pasteurisation treatment group (Table 3).

**Table 3:** One-way ANOVA summary table for the four-point bending compressive test of the sheep tibial bone with plate osteosynthesis treated with cryotherapy and pasteurisation group.

Parameter		df	Sum of Squares	Mean Square	F	Sig.
Maximum Compressive Load (N)	Between Groups	2	55120.083	27560.041	0.161	0.853
	Within Groups	15	2571300.222	171420.015		
	Total	17	2626420.305			
Compressive Stress (MPa)	Between Groups	2	25.292	12.646	0.603	0.560
	Within Groups	15	314.563	20.971		
	Total	17	339.855			
Compressive Strain (mm)	Between Groups	2	31.781	15.891	1.955	0.176
	Within Groups	15	121.904	8.127		
	Total	17	153.686			

Photographic documentation of failure patterns was also recorded. In the control group, all points of failure were seen to have originated from the screw holes at the distal part of the sheep tibia, with the fracture starting from the first and second holes from the osteotomy site. This is likely due to the fact that it is the closest site to the superior jig compression points. There was no fracture originating from the screw holes of the proximal tibia. As the sheep tibia bone is not uniform in shape and the diameter at the proximal tibia is larger, it provides more purchasing bone stock to the screws. No screw or plate fracture occurs in the control group.

**Figure 6:**
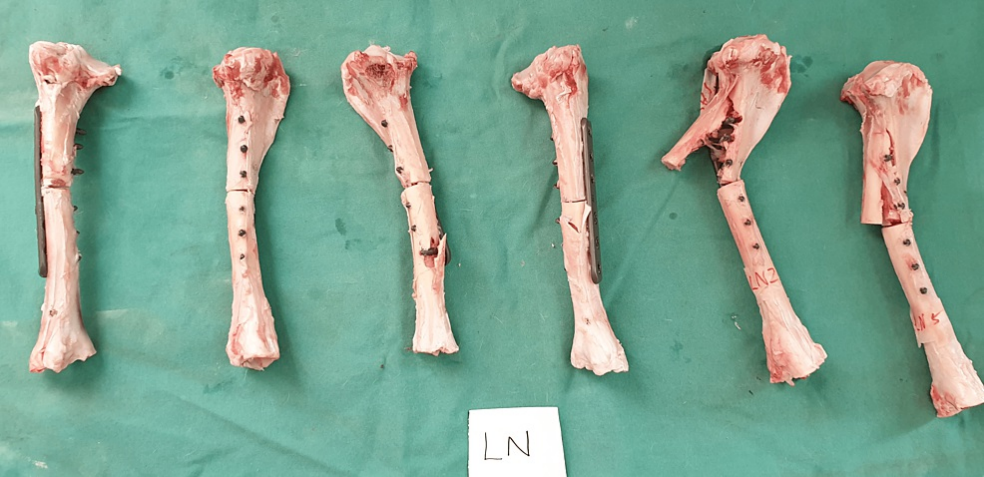
Individual fracture pattern for each of the specimens in the cryotherapy group.

**Figure 7:**
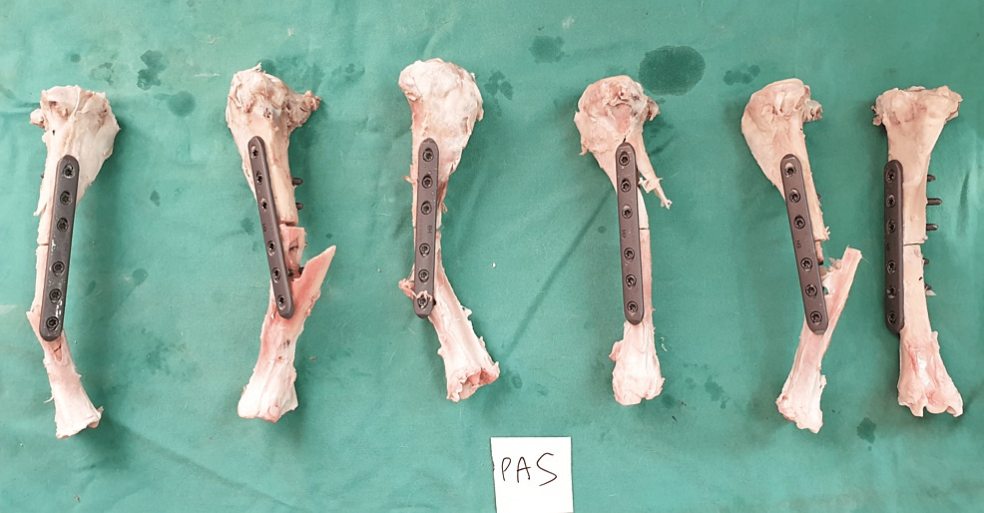
Individual fracture pattern for each of the specimens in the pasteurisation group.

For the liquid nitrogen-treated sheep tibia, all failure points occurred at the screw holes, representing the weakest point of the entire construct. Fractures at the screw hole occurred at the distal sheep tibia for three of the specimens, three fractures started at the proximal fragment, sheering through all three proximal screw holes (Figure 6). Pasteurized specimens demonstrated five fractures starting at the distal part of the sheep tibia and one fracture starting at the proximal part of the construct (Figure 7).

In all groups, the locking system of the plate and screw remained intact. There was no incidence of loosening of the screw from the locking thread and there was also no back out of the screw from the plate.

## Discussion

Osteosarcoma remains a severely debilitating pathology, especially in young adults. Common treatment includes chemotherapy, radiotherapy, and limb salvage surgery [1]. Limb reconstruction of the resected bone using endoprosthesis, allograft, or autograft is among the options [7,21]. Autologous bone graft remains an option based on their biological component and availability in certain cases. For the autologous bone graft to succeed, malignant cells must be eradicated from the resected bone. To achieve this, several methods have been described, namely cryotherapy with liquid nitrogen, boiling, pasteurization, autoclave, and gamma irradiation which have been proven to sterilize the bone from tumour cells [11,24,28].

A titanium locking plate was used in our study as it provides better strength, eliminates the need for perfect bone contact between the plate and the bone surface, and is a commonly used implant in clinical practice. In the principle of tibia plating, four screws are needed to be inserted in each part (distal and proximal) of the fracture [29]. However, due to the length of the sheep tibia, the six-hole plate was a better fit. Standard handling and fixing of the implant were performed on the bone to replicate the condition when a plating procedure is done on a human bone to minimize the disruption of the biology of the bones.

In our study, there was no statistical significance in all three biomechanical property parameters of the reconstructed sterilized bone graft using liquid nitrogen (cryotherapy) and pasteurization technique, in comparison to the control group. This shows that both sterilization methods give comparable biomechanical properties as untreated bone and none is superior to the other. This finding for liquid nitrogen was quite similar to a study by Yamamoto et al. that reported that there was no significant difference in compression strength between intact bone and liquid nitrogen-treated bone, whereas the strength of the autoclaved bone was decreased [25]. In a study by Singh et al., it was reported that methods involving high temperature, which include boiling and autoclaving, degrade the biomechanical property of the bone, pasteurization degrades the bone to a lesser extent, and irradiation does not alter the biomechanical property of the bone [24]. On the other hand, Yassin et al. in 2015 reported differently in an experiment using rabbits, where they showed that in all sterilized bone grafts using autoclave, irradiation, and pasteurisation, the mechanical strength dropped significantly compared to the control group at 12 weeks and pasteurized autogenous bone graft had the lowest mechanical strength; when the sterilized groups were compared, autoclaved bone graft generally had a higher maximum load, stress to failure, and strain to failure compared to pasteurisation and irradiation but this was not statistically significant at six, nine and 12 weeks [11]. Differences in methodology and specimen type may have been the cause of the difference in our results.

All of the construct failures occurred at the screw holes. In the experimental group, three fractures in the liquid nitrogen group and one fracture in the pasteurisation group (four of the 12 samples; 33.3%) were seen to be originating from the metaphyseal region of the proximal tibia. Although the proximal screws were mainly inserted into the metaphyseal region of the tibia, fewer fractures occurred in this region compared to the distal diaphysis region despite the fact that metaphyseal bones are known to be more porous than diaphyseal bones. These findings, however, agree with the results of several clinical studies reporting on the failure of locking plate osteosynthesis, which is mostly located within the zone proximal to the fracture in relation to the distal femur, i.e. the diaphysis [30]. We postulated it may be due to the metaphyseal region of the tibia being significantly larger in diameter compared to the diaphyseal region and longer screw lengths with more bone purchase can be used. Looking at the biomechanical properties results and pattern of the fracture, the sterilization technique can be proved not to cause weakening of the bone. Other than that, there was no plate or screw fracture in this study; hence, a plate with a locking system seems to be a suitable and stable implant to be used, relative to the strength of the bone.

Although both pasteurisation and liquid nitrogen sterilization technique were shown to provide comparable initial structural and mechanical property post treatment, decisions on which method to use has to be determined based on the local settings, availability of the facility needed, and personnel familiarity with each of the methods. As an example, a proper protocol has to be established for the safe handling and usage of liquid nitrogen, proper storage, procedure room, and personal protective equipment as it can cause severe thermal injury upon skin or eye contact. Pasteurisation might present itself as a more accessible method as a water bath is generally safer to use and more readily available.

In clinical settings, resected bones from the patient are not healthy bones, commonly with defects caused by underlying tumours. Diseased bones might not have similar properties in comparison to healthy bones and the biomechanical properties of said bones might produce a different result after being subjected to the sterilization methods, which is another point to be considered.

The experiment was done on a cadaveric sheep bone. Even though efforts were taken to preserve the biology of the bone to the greatest extent, the results of the experiment only serve to demonstrate the immediate strength and biomechanical properties of the bone segments treated with the sterilization method. Bone healing requires time and surrounding biological envelopes to heal. Sterilization eradicates tumour cells and healthy cells, including osteocytes indiscriminately. The union of the bone and future biomechanical property of the bone post implantation into the patient is yet to be determined. The revitalisation of the treated bone by surrounding soft tissue has to occur in order for a union to occur. Resorption of non-viable or non-revitalised tissue has to be taken into consideration. These factors might affect the future stability and strength of the reconstruction limb, especially when the patient is allowed weight-bearing ambulation.

## Conclusions

There was no statistically significant difference in the biomechanical properties, namely maximum compression load, compressive stress, and strain, of the bone treated with cryotherapy and pasteurization. Both methods did not cause significant degradation of bone strength as compared to the control group. However, this only illustrates the immediate strength and stability of the bone post sterilization. More in vivo studies are needed to highlight the long-term effects and outcomes of the sterilization method.
